# The underlying mechanisms of the association of bone health with depression – an experimental study

**DOI:** 10.1007/s11033-025-10230-x

**Published:** 2025-01-27

**Authors:** Sanne Paulien Houtenbos, Yangyang He, Petra Cazzanelli, George Soultoukis, Karin Wuertz-Kozak, Tim J. Schulz, Pia-Maria Wippert

**Affiliations:** 1 Medical Sociology and Psychobiology, Department of Health and Physical Activity, University of Potsdam, 14469 Potsdam, Germany; 2 Department of Biomedical Engineering, Rochester Institute of Technology (RIT), Rochester, NY 14623 USA; 3 Spine Center, Schön Klinik München Harlaching, Academic Teaching Hospital and Spine Research Institute of The Paracelsus Private Medical University Salzburg (Austria), 81547 Munich, Germany; 4 Faculty of Health Sciences Brandenburg, Joint Faculty of the University of Potsdam, The Brandenburg, Medical School Theodor Fontane and The Brandenburg University of Technology Cottbus—Senftenberg, 14469 Potsdam, Germany; 5 Department of Adipocyte Development and Nutrition, German Institute of Human Nutrition Potsdam-Rehbrücke, 14458 Nuthetal, Germany; 6 German Center for Diabetes Research (DZD), 85764 München-Neuherberg, Germany; 7 Institute of Nutritional Science, University of Potsdam, 14558 Nuthetal, Germany

**Keywords:** Bone turnover markers, Molecular mechanisms, Stress-related disorders

## Abstract

**Background:**

Depression constitutes a risk factor for osteoporosis, but underlying molecular and cellular mechanisms are not fully understood. MiRNAs influence gene expression and are carried by extracellular vesicles (EV), affecting cell-cell communication. Aims: (1) Identify the difference in miRNA expression between depressed patients and healthy controls; (2) Analyze associations of these miRNAs with bone turnover markers; (3) Analyze target genes of differentially regulated miRNAs and predict associated pathways regarding depression and bone metabolism.

**Methods and results:**

Blood samples from depressed patients (*n* = 11) were obtained from a previous study and healthy controls (*n* = 9) were recruited. Sociodemographic, depression diagnosis and depressive symptom (BDI-II) data were collected through questionnaires. Blood plasma was collected from each participant and real-time-quantitative PCR was performed on isolated plasma EVs; differences in miRNA expression between groups were analyzed using qbase+. Regression models assessed the associations of differentially regulated miRNAs with bone turnover markers procollagen-1 N-terminal-peptide, osteocalcin, and crosslaps; enriched pathways and miRNA target gene networks were analyzed. 19 miRNAs were differentially expressed between groups (*p < 0.05*). MiR-26b-5p and miR-106a-5p showed an association with procollagen-1 N-terminal-peptide; miR-330-5p and miR-377-3p were associated with osteocalcin, and miR-26b-5p, miR-34c-3p and miR-145 with crosslaps. Pathway analysis including the differentially expressed miRNAs predicted enriched pathways, including the FoxO signaling and p53 signaling pathway. Seven target genes were identified.

**Conclusions:**

MiRNAs (e.g. miR-26b-5p, miR-377-3p), genes (TNRC6B, HSPA8), and pathways (FoxO- and Hippo-signaling pathway) are identified which could be mediators between the influence of depression on bone health and could possibly serve as biomarkers in the treatment of bone diseases among people with mental disorders.

**Supplementary Information:**

The online version contains supplementary material available at 10.1007/s11033-025-10230-x.

## Background

Due to a worldwide aging population, age-related pathologies become more frequent. Osteoporosis (OP) is such a pathology and can be divided into two types: primary (due to aging) and secondary (caused by diseases or treatments) [1]. The worldwide prevalence of OP is estimated to be 18.3% based on 86 studies, depending on geographical location, with the highest prevalence estimated to be in Asia and Africa [1]. Among many other factors, depression constitutes a risk factor for OP [2], and people with osteoporosis have shown higher levels of depression compared to people who are not affected by this pathology [3]. Similarly, depression is a highly prevalent disease among the global population with high disability and low complete remission rates [3, 4].

Although depression and OP are on numerous occasions simultaneously present in patients, the mechanisms behind the influence of depression on bone health, and its eventual role in the development of OP, are not completely understood. It is known that depression, clinically named Major Depressive Disorder (MDD), is a stress-related disorder that can lead to a dysregulation of the Hypothalamic-Pituitary-Adrenal (HPA) axis [3–5]. This dysregulation has many negative consequences, such as an altered expression of growth hormones (e.g. IGF-1), and an increased release of inflammatory cytokines and glucocorticoids [2, 5, 6]. The release of pro-inflammatory cytokines induces osteoclast activation, and glucocorticoids and IGF-1 further affect anabolic bone metabolism and density, resulting in an imbalance in osteoblast and osteoclast activity and hence, a potential decrease in bone mineral density (BMD) [7–9].

Most research regarding the association between depression and bone health is conducted based on the outcome BMD (obtained through DEXA scans), but the molecular and cellular processes underlying the changes in BMD have not been well researched until now. Recent studies have highlighted the changes in cellular and mitochondrial activity among groups with mental and stress-related disorders, including depression [10]. It is known that, at a cellular level, extracellular vesicles (EV) are important carriers in this cell-to-cell communication [11]. EVs contain crucial genetic and cell-regulating information, such as DNA, RNA, and other proteins and metabolites [11]. EVs also contain miRNAs, which are non-coding RNAs that influence gene expression [11], by binding to and targeting mRNAs [12]. Depressed patients have an altered expression of certain miRNAs compared to people without depression [13], which could in turn have an influence on bone metabolism. A review by Ding et al. (2023) highlighted the role of miRNAs in MDD and their potential as biomarkers for depression treatment [13]. Similarly, Gao, Patil & Qian (2020) focused on the role of miRNAs in bone metabolism [14]. Combining this knowledge and identifying specific miRNAs that are dysregulated due to depression, and their role in bone metabolism, is of interest. In the future, these miRNAs could be used as diagnostic markers to assess treatment effectiveness for osteoporosis.

Therefore, this paper aimed to discover the potential role of miRNAs (in plasma EVs) as mediators between depression and bone metabolism. To achieve this, we first assessed differences in EV miRNA expression between depressed patients and healthy controls. Secondly, we focused on these differentially regulated miRNAs and calculated associations between their expression and levels of bone markers (in plasma). These bone markers included procollagen type 1 N-propeptide (P1NP), osteocalcin (OC), both markers of bone formation, and crosslapps (CTx) which is a marker of bone resorption. Lastly, we used the open access software to identify miRNA pathways and target genes and identify those relevant in the context of depression and bone health.

## Materials and methods

### Participants

Data and blood samples of two groups were used for the purpose of this paper: blood samples and questionnaire data of depressed patients stemming from a previous interventional study (DEPREHA, *n* = 11 [8]) and from newly recruited healthy control participants (*n* = 9). The recruitment of depressed participants was conducted according to the following inclusion criteria: aged 18–65 years, depressive episode (ICD-10 F32.x or F33.x) diagnosis, dysthymia (F34.1), or adjustment disorder with a prolonged depressive reaction (F43.21), inability to work for $\:\text{≥}$21 days during the past 12 months due to the diagnosis mentioned above. The inclusion criteria for the healthy controls were that they should not be suffering from any mental pathologies and aged 18–65 years. Exclusion criteria for both groups consisted of pregnancy, hormonal therapy (excluding hormonal contraception), intellectual disability (ICD10 F70-89), compliance with other primary diagnoses (e.g., hormonal/endocrine metabolic disorders (diabetes mellitus, thyroid dysfunction, renal, hepatic disorders, etc.), neurological disorders; dementia (ICD-10 F00-F03), psychotropic drug dependence syndrome (ICD-10 F1x.2), schizophrenia (ICD-10 F20), psychotic, stress, and somatoform disorders (F40-49, unless they fall within the inclusion criteria), emotionally unstable personality disorder (ICD-10 F60.3x), as well as other personality and behavioral disorders (F61-F69), acute infections, immune system disorders, unstable remitting addictions, and acute drug abuse (excluding nicotine).

All participants in this experiment were informed of the purpose and content of the study both verbally and in written form, and all participants signed the informed consent form. The clinical investigations were conducted according to the principles of the Declaration of Helsinki. Final ethical approval was obtained on 11.05.2021 from the Ethics Review Board of the University of Potsdam, Germany (number 19/2021).

### Data collection

#### Psychometric measures

Sociodemographic data (e.g., age, gender, socioeconomic status, etc.) were collected through questionnaires. A clinical diagnosis of depression was collected from the depressed group. Furthermore, the Beck Depression Inventory-II (BDI-II) questionnaire [15] was used to assess depressive symptoms and severity in both groups. The BDI-II is a 21-item self-reported questionnaire that addresses current affective, cognitive, motivational, and physiological symptoms of depression, answered on a 4-point Likert scale. The internal consistency of the BDI-II questionnaire was Cronbach’s alpha 0.96.

#### Plasma samples collection

Ten milliliters of blood were drawn from each participant (between 7 and 9 am) into EDTA blood tubes (Sarstedt, Germany). As preparation, the participants were instructed to not eat anything in the 12 h before the blood was drawn and to only drink water. Additionally, they were instructed to not take unscheduled or heavy medication, and to refrain from consuming high amounts of tea, coffee, or do intense exercise in the 24 h before. The blood was left standing vertically at 4 °C for 30 min and afterwards centrifuged at 1500 g for 20 min to retrieve the platelet-poor plasma [16], which was utilized for EV isolation. The EDTA-plasma was aliquoted in 500 µL tubes and stored in a -80 °C freezer for future use.

#### EV Harvest

EVs from the plasma were isolated with Systems Bioscience’s thrombin and ExoQuick solutions (SBI, USA). Prior to the routine steps, the plasma was centrifuged at 3000 g for 15 min to remove cell debris. To remove fibrin, 5 µl Thrombin (SBI, USA) was added to 500 µl of plasma and incubated at room temperature for 5 min, and afterwards centrifuged at 10,000 rpm for 5 min. To finally isolate EVs, 120 µl ExoQuick was added to the serum-like supernatant, incubated for 30 min at 4 °C, and then centrifuged at 13,000 rpm for two minutes.

#### EV RNA isolation

To isolate RNA, the SeraMir Kits (SBI, USA) were used. The supernatant was removed and 350 µl LYSIS buffer was added. Next, 200 µl 100% ethanol (PanReac AppliChem, Germany) was added to the sample, the mixture was transferred to the spin column and centrifuged at 13,000 rpm for one minute. Afterwards, 400 µl wash buffer was added and centrifuged at 13,000 rpm for one minute (repeated twice). Lastly, the collection tube was thrown out, and the spin column was assembled with a 1.5 ml Eppendorf tube. 30 µl elution buffer was added to the spin column and centrifuged at 2000 rpm, for two minutes to load buffer, then increased to 13,000 rpm for one minute. To assess the quality and quantity of the isolated EV RNA, the Agilent 2100 Bioanalyzer and RNA 6000 Pico chip Kits (Agilent Technologies, USA) were used; the average yield from 500 µl plasma was 1–10 ng of total RNA. Samples below this concentration were discarded, and EV RNAs above this concentration were diluted with RNase-free water to the appropriate concentration for subsequent experiments. The eligible EV RNAs, based on the criteria mentioned above, were selected for use in subsequent experiments.

#### EV RNA reverse transcription and miRNA real-time quantitative PCR

Total RNA, 5 µl, was reverse transcribed (cDNA synthesis) using SeraMir Kits (SBI, USA) according to the manufacturer’s instructions. Real-time quantitative PCR (qPCR) was performed with a 384-well SeraMir Profiler with the 2X Maxima SYBR Green/ROX qPCR Master Mix (Thermo Scientific, USA) on the CFX384 Touch Real-Time PCR Detection System (Bio-Rad Laboratories, USA) and 2X Maxima SYBR Green/ROX qPCR Master Mix (Thermo Scientific, USA) with the appropriate temperature cycles 50 °C/2 min, 95 °C/10 min, 40 cycles; 95 °C/15 s, 60 °C/1 min; data read at 60 °C/1 min. The miRNA expression values were calculated using global mean normalization to reduce the impact of batch effects.

#### Bone turnover markers

The expression of bone turnover markers Procollagen type 1 N-propeptide (P1NP), osteocalcin (OC), and crosslapps (CTx) was analyzed by the Ernst von Bergmann Lab in Potsdam, Germany, using the electrochemiluminescence immunoassays “ECLIA” from Roche COBAS Elecsys 2010 MODULAR ANALYTICS E170 (REF 03141071 190 for P1NP; REF 12149133 for OC; REF 11972308 122 for CTx (F. Hoffmann-La Roche, Ltd., Basel, Switzerland).

P1NP is a peptide mainly stemming from proliferating osteoblasts and fibroblasts, and utilized as a marker of bone formation [17, 18]. Osteocalcin is a protein synthesized by osteoblasts, hypertrophic chondrocytes, and odontoblasts, shown to be highly correlated with an increase in BMD during treatment for OP. At the same time, OC fragments are released during bone resorption [19] and therefore used as a marker of bone turnover instead of solely bone formation [17–19]. CTx is a degradation product of type 1 bone collagen and a marker of bone resorption [17, 18].

### Analysis

#### Identification of differentially regulated EV miRNAs

The miRNA expression was calculated according to the 2^−∆∆Ct^ method, where the expression of the depressed group was calculated relative to the healthy control group. The miRNA expression values were calculated using the three endogenous controls. Differences in miRNA expression were analyzed using Mann-Whitney-U tests. The p-value was adjusted using the FDR multiple comparison, according to the Benjamini and Hochberg method [20]. The thresholds for significantly different expressed miRNAs were log_2_ fold change > 1 or <-1 with adjusted p-values (FDR) < 0.05 using the software qbase+.

#### Prediction of depression-related pathways

DNA Intelligent Analysis (DIANA)-miRPath v4.0 software was used to identify the enriched pathways related to both up-regulated and down-regulated miRNAs between depressed patients and healthy controls. This software identifies the targeted “Kyoto Encyclopedia of Genes and Genomes (KEGG)” for systematic analysis of miRNA functions, linking genomic information with higher order functional information. The KEGG pathway database (https://www.genome.jp/kegg/pathway.html) was used to classify the category of miRNA-related pathways. The “pathways union” option of the miRPath software was used. P-values were obtained by the Fisher’s exact test as an enrichment analysis method, and the false discovery rate (FDR) was estimated using the Benjamini and Hochberg method [20]. Cancer-related pathways were deleted from our analysis, since there is a strong bias in the direction of these pathways compared to others in the database.

#### Predicting the target genes of differently expressed miRNAs

The databases TargetScan, miRtarbase, and miRDB were selected for miRNA target gene prediction, and the intersection of the three databases was taken by Venn as the target gene for miRNA prediction in order to reduce the false positive rate. The results from the three databases were imported into the Cytoscape software, which was used to construct and visualize the miRNA-mRNA interaction. In the diagram made in Cytoscape, each node represents a miRNA or mRNA, and the edge between nodes represents the interaction between miRNAs or mRNAs. The most associated target mRNAs, which were extracted by cytoHubba (version 0.1), are often highly correlated with other miRNAs and virtual nodes in biological networks.

#### Statistics

The data collected from the questionnaires were prepared along psychometric manual rules. The data analyses were conducted using the IBM SPSS Statistics program (IBM SPSS 23.0). Descriptive data were presented as median ± interquartile range. Differences between groups were analyzed with the Mann-Whitney U-test or the Chi-Square test. General linear regression analyses were performed to assess the association between selected miRNAs and bone markers, controlling for age and sex. Three models were constructed: Model 1 was unadjusted; Model 2 was adjusted for age and sex, and Model 3 was adjusted for BMI.

## Results

### Participant characteristics

A total of *n* = 20 participants (*n* = 11 depressed; *n* = 9 healthy controls) were included in the presented analysis. Table 1 shows the group characteristics for the depressed patients, healthy controls and both combined. The overall (*n* = 20) median age was 40.55 (IQR: 13.77), 60% were female and the BDI-II score (*n* = 18) was 12.00 (IQR: 21.00). There were significant differences for age (47.27 (11.46) vs. 32.33 (12.18) y, BMI (27.10 (5.08) vs. 21.40 (2.30) kg/m^2^, BDI-II (12.00 (23.00) vs. 5.50 (9.00)) and CTx (0.36 (0.38) vs. 0.58 (0.07) ng/mL between groups.

**Table 1:** Descriptive characteristics (median and interquartile range (IQR)) of the overall sample, and the depressed and healthy control group separately

Variable	*N*	All	*N*	Depressed	*N*	Controls	*P*-value
Sex (M/F)	20	8/12	11	4/7	9	4/5	0.731
Age (y)	20	40.55 ± 13.77	11	47.27 ± 11.46	9	32.33 ± 12.18	0.011*
BMI (kg/m^2^)	19	25.08 ± 7.30	11	27.10 ± 5.08	8	21.40 ± 2.30	0.012*
Smoking (n/y)	20	17/3	11	8/3	9	9/0	0.089
**Depression**							
BDI-II	18	12.00 ± 21.00	10	21.00 ± 23.00	8	5.50 ± 9.00	0.001**
**Bone markers**							
P1NP (ng/mL)	19	54.00 ± 25.90	11	43.80 ± 30.30	8	57.70 ± 18.22	0.062
OC (ng/mL)	18	16.25 ± 6.55	11	17.40 ± 7.70	7	13.20 ± 5.40	0.375
CTx (ng/mL)	19	0.56 ± 0.29	11	0.36 ± 0.38	8	0.58 ± 0.07	0.026*

N: number of samples; y: years; kg/m^2^: kilogram per square meter; n/y: no/yes; *p < 0.05; **p < 0.01

### Altered miRNA expression between depressed patients and healthy controls

**Fig. 1:**
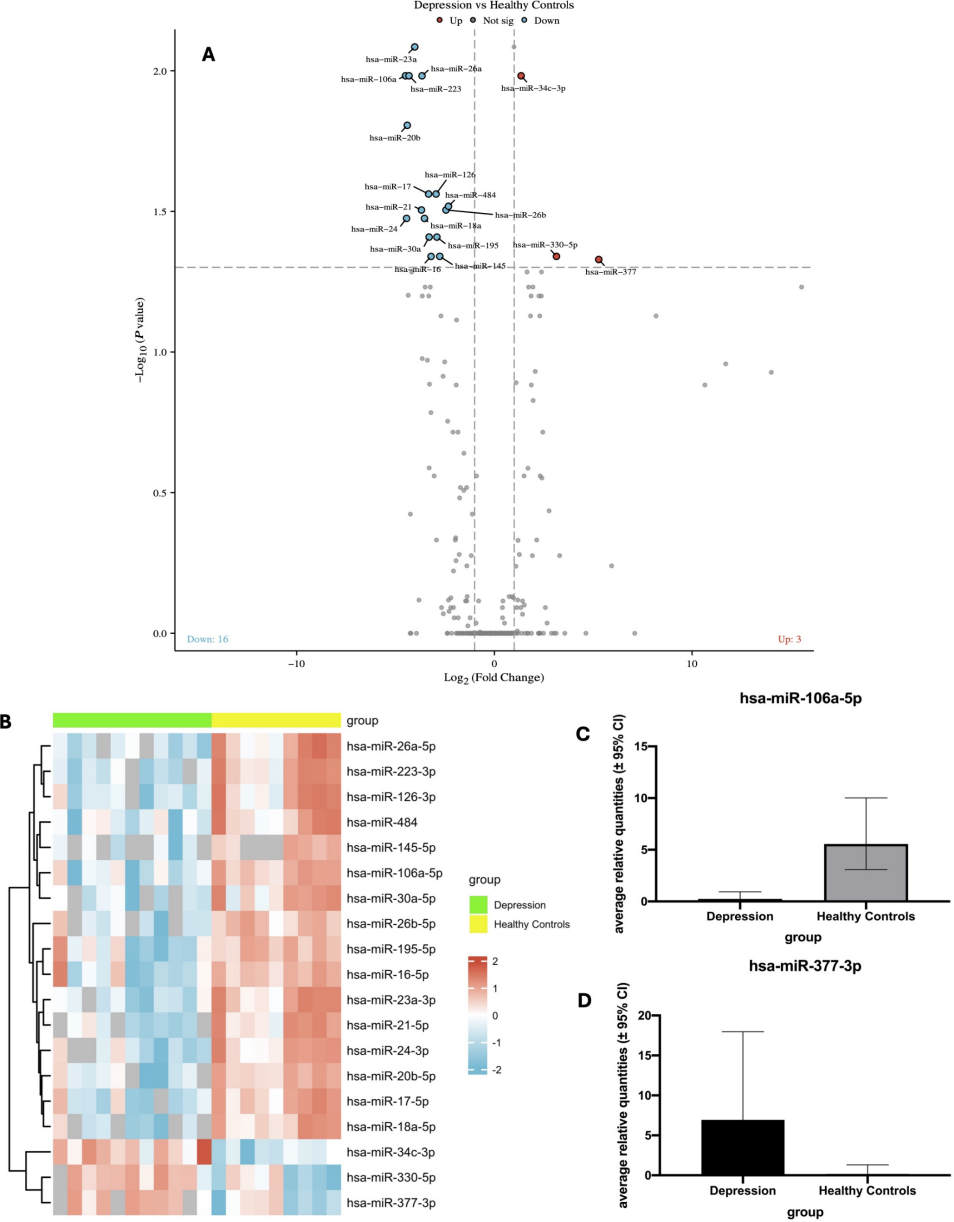
Volcano plot (**A**) and Heatmap (**B**) of the significant differences (2-fold regulation change; *p* < 0.05) in miRNA expression between depressed patients and healthy controls, bar graphs of the highest down-regulated (**C**) and up-regulated (**D**) miRNAs

Overall, 19 miRNAs were discovered with significant differences in expression between depressed patients and healthy controls (Fig. 1). Sixteen miRNAs (miR-16-5p, miR-17-5p, miR-18a-5p, miR-20b-5p, miR-21-5p, miR-23a-3p, miR-24-3p, miR-26a-5p, miR-26b-5p, miR-30a-5p, miR-106a-5p, miR-126-3p, miR-145-5p, miR-195-5p, miR-223-3p, and miR-484. were significantly downregulated and three (miR-34c-3p, miR-330-5p, and miR-377-3p) were upregulated in the depressed group compared to the controls. The differences in expression between groups for miR-106a-5p and miR-377-3p are shown in Fig. 1 as examples, the graphs for the remaining miRNAs are included in the supplementary material.

### Association of miRNAs with bone turnover markers

Table 2 shows the associations of the six differentially regulated miRNAs that had a significant association with one or more of the three analyzed bone turnover markers (P1NP, OC, CTx). Associations are highlighted in blue. P1NP was associated with miR-26b-5p (*p < 0.01* (model 1); *p* < 0.05 (model 2 and 3)) and miR-106a-5p (*p < 0.05* (model 1)). OC was associated with miR-330-5p (*p < 0.01* (model 2 and 3)) and miR377-3p (*p < 0.05* (model 1, 2 and 3). CTx was associated with miR-26b-5p (*p < 0.05* (model 1)), miR-34c-3p (*p < 0.01* (model 1); *p < 0.05* (model 2)) and miR-145-5p (*p < 0.05* (model 1)).

**Table 2:**
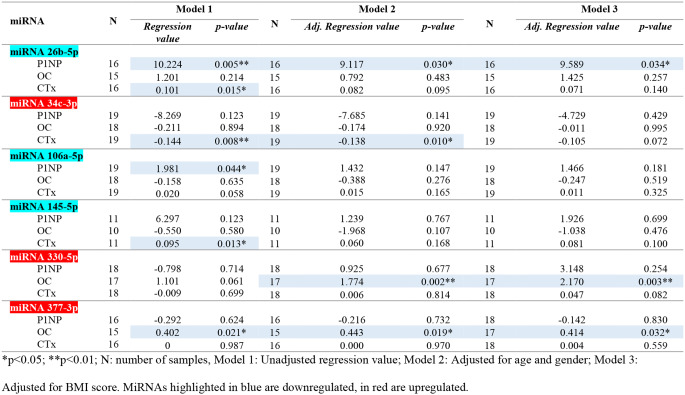
Linear regression of the significant associations with bone health of the six miRNAs with significant differences between the depressed vs. healthy control group

### miRNA pathways and target genes

In Table 3, the top 10 most enriched pathways based on miRNA number, according to the DIANA-miRPath v4.0 software, are shown. Four pathways were attributed to cellular processes, two were related to environmental information processing, two other pathways to human diseases, one to genetic information processing, and lastly one to organismal systems.

**Table 3:** The top 10 most enriched pathways related to the 19 human miRNAs with an altered expression between the depressed and healthy control groups

KEGG Pathway	Term name	miRNAs (*N*)	miRNAs	Merged FDR
Environmental information processing: signal transduction	FoxO signaling pathway	13	miR-16-5p, miR-17-5p, miR-21-5p, miR-23a-3p, miR-24-3p, miR-26a-5p, miR-26b-5p, miR-106a-5p, miR-223-3p, miR-126-3p, miR-195-5p, miR-377-3p, miR-20b-5p	5.13^− 48^
Cellular processes: cellular community–- eukaryotes	Focal adhesion	13	miR-16-5p, miR-17-5p, miR-21-5p, miR-23a-3p, miR-24-3p, miR-26a-5p, miR-26b-5p, miR-106a-5p, miR-145-5p, miR-195-5p, miR-377-3p, miR-20b-5p, miR-484	5.13^− 48^
Cellular processes: cell growth and death	p53 signaling pathway	13	miR-16-5p, miR-17-5p, miR-18a-5p, miR-21-5p, miR-23a-3p, miR-24-3p, miR-26a-5p, miR-26b-5p, miR-30a-5p, miR-145-5p, miR-195-5p, miR-20b-5p, miR-484	4.55^− 42^
Cellular processes: cell growth and death	Cell cycle	12	miR-16-5p, miR-17-5p, miR-18a-5p, miR-21-5p, miR-23a-3p, miR-24-3p, miR-26a-5p, miR-26b-5p, miR-30a-5p, miR-195-5p, miR-20b-5p, miR-484	1.66^− 44^
Human diseases: infectious diseases: bacterial	Shigellosis	12	miR-16-5p, miR-17-5p, miR-21-5p, miR-23a-3p, miR-24-3p, miR-26a-5p, miR-26b-5p, miR-106a-5p, miR-145-5p, miR-126-3p, miR-195-5p, miR-20b-5p	1.61^− 34^
Cellular processes: cell growth and death	Cellular senescence	11	miR-16-5p, miR-17-5p, miR-23a-3p, miR-24-3p, miR-26a-5p, miR-26b-5p, miR-30a-5p, miR-106a-5p, miR-126-3p, miR-195-5p, miR-20b-5p	6.08^− 26^
Genetic information processing: folding, sorting, and degradation	Ubiquitin mediated proteolysis	10	miR-16-5p, miR-17-5p, miR-18a-5p, miR-23a-3p, miR-26a-5p, miR-26b-5p, miR-30a-5p, miR-106a-5p, miR-195-5p, miR-20b-5p	1.81^− 36^
Environmental information processing: signal transduction	Hippo signaling pathway	10	miR-16-5p, miR-17-5p, miR-18a-5p, miR-24-3p, miR-26a-5p, miR-26b-5p, miR-30a-5p, miR-106a-5p, miR-145-5p, miR-195-5p	1.04^− 27^
Organismal systems: endocrine system	Thyroid hormone signaling pathway	10	miR-16-5p, miR-17-5p, miR-21-5p, miR-23a-3p, miR-24-3p, miR-26a-5p, miR-26b-5p, miR-30a-5p, miR-145-5p, miR-20b-5p	1.62^− 27^
Human diseases: infectious disease: bacterial	Salmonella infection	10	miR-16-5p, miR-17-5p, miR-18a-5p, miR-23a-3p, miR-24-3p, miR-26a-5p, miR-30a-5p, miR-106a-5p, miR-145-5p, miR-195-5p	1.38^− 27^

KEGG: Kyoto Encyclopedia of Genes and Genomes, N: number of samples, FDR: false discovery rate

**Fig. 2:**
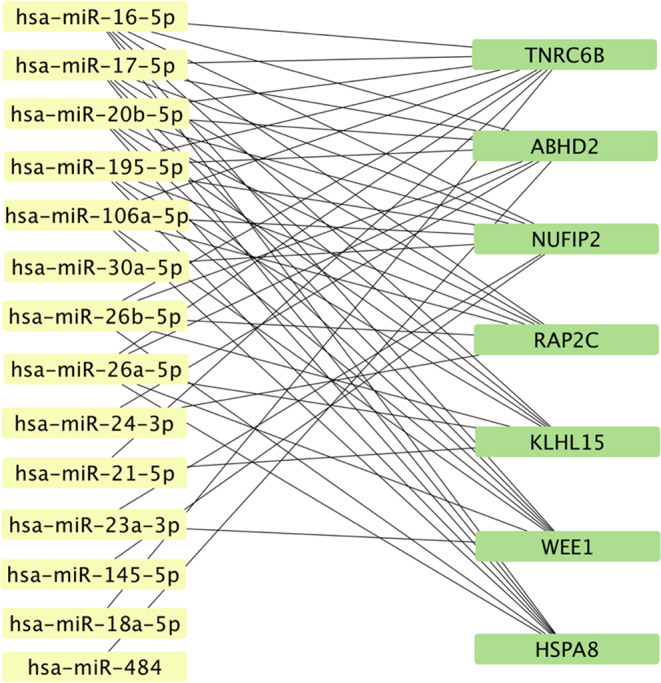
The target mRNAs most associated with the differently expressed miRNAs in the depressed patients group generated with the Cytoscape software

Using Cytoscape, a miRNA-mRNA interaction network was created, based on the target gene predictions of Targetscan, miRtarbase, and miRDB. Fourteen out of the nineteen differentially expressed miRNAs were associated with specific target mRNAs. According to the analysis, the most associated target mRNAs of these differently expressed miRNAs in the depressed patients group, listed in sequence, were Trinucleotide Repeat Containing Adaptor 6B (TNRC6B), Abhydrolase domain-containing protein 2 (ABHD2), Nuclear FMR1 interacting protein 2 (NUFIP2), Ras-related protein RAP-2c (RAP2C), Kelch Like Family Member 15 (KLHL15), WEE1 G2 checkpoint Kinase (WEE1), Heat Shock Protein Family A (Hsp70) Member 8 (HSPA8), shown in Fig. 2.

## Discussion

The current paper aimed to investigate the relationship between depression and bone health on the molecular biological level. EVs have gained increasing attention for their role in intercellular communication and disease pathogenesis. The expression of miRNAs, also known as epigenetic regulatory mechanisms, in plasma EVs were compared between depressed and healthy subjects in this study. Overall, multiple miRNAs were detected and predicted to target several pathways and genes which seem to play a role in the negative association of depression with bone metabolism.

In total, 19 out of 380 miRNAs were differentially expressed between depressed patients and healthy controls. Some of these 19 miRNAs have been suggested as possible biomarkers for depression in previous studies. According to a systematic review from Ferrúa et al. (2019), regarding the expression of miRNAs among depressed patients, miR-17-5p was one of the most frequently detected miRNAs among depression-related pathways [21]. Further, miR-223-3p levels at baseline have been used as a predictor for responsiveness to electroconvulsive therapy [22]. Interestingly, the miRNA expression results among depressed patients detected in the current study are mostly in line with other studies. For instance, the expression of miR-16-5p is downregulated in MDD in other studies, including in the current project (fold change: 0.11; *p* = 0.046) [23, 24]. Similarly, miR-145 was downregulated in another study (upregulated after treatment [25]), while it was also downregulated in the current analysis (fold change: 0.15; *p* = 0.046). The analyses performed in the current project were explorative, meaning that there was no focus on specific miRNAs. Future studies should validate these identified miRNAs with a potential role in depression pathogenesis and their function as biomarkers for depression in a larger sample set.

For the identified differentially expressed miRNAs, we also analyzed their association with bone health (i.e. bone turnover marker levels) in the current project. Three linear regression models were applied: Model 1: unadjusted; Model 2: controlled for age and sex, Model 3: controlled for BMI. Controlling for age and BMI was carried out since significant differences existed between groups for these variables. Six out of the 19 miRNAs that were differentially expressed in the depressed group showed an association with at least one of the three bone markers (P1NP, OC and CTx). The bone formation factor P1NP was positively associated with two miRNAs: miR-26b-5p and miR-106a-5p. The association of miR-26b-5p with P1NP was significant both when unadjusted and when correcting for age and sex, or BMI. Previous studies have noted that the expression of miR-26b is upregulated in BMSCs during osteogenesis [26] and miR-26b-5p is one of the miRNAs that regulates SMAD1, a core mediator of bone morphogenetic protein (BMP) signaling that regulates osteogenic differentiation [27]. As we found miR-26b-5p to be significantly downregulated with depression and to show a positive association with P1NP, which is a marker for bone formation, this would result in a downregulation of bone formation. The association of miR-106a-5p with P1NP was not significant anymore after controlling for age and sex, or BMI, meaning that this association was influenced by these confounding factors. Even though this association is influenced by additional factors, miR-106a-5p has been shown to suppress osteogenic differentiation and simultaneously advance adipogenic differentiation of human adipose-derived mesenchymal stem cells [28]. Contrastingly to what was found for miR-26b-5p, a downregulation of this miRNA with depression could indicate a tendency to increased bone differentiation. Depressed and healthy groups were not matched regarding participant characteristics; there were baseline differences between groups for age, BMI (due to weight) and alcohol consumption. By correcting for age and sex, and BMI, it was attempted to assess the influence of these confounding factors on the association between miRNAs and bone biomarkers.

We also investigated associations of miRNAs with OC, a marker of bone turnover. OC is released into the circulation from the matrix during bone resorption and, therefore, is considered a marker of bone turnover rather than a specific marker of bone formation, i.e. high levels of OC point to bone loss and predict low bone density and fracture risk. OC was positively associated with two miRNAs: miR-330-5p and miR-377-3p, both of which were upregulated with depression. Li et al. (2021) saw that miR-330-5p attenuated osteogenic differentiation in BMSCs and pre-osteoblasts, and silencing miR-330-5p led to an upregulation of osteogenesis in BMSCs [29]. Similarly, Jin et al. (2020) silenced the expression of miR-330-5p in osteoporotic mice, which led to an increase of genes related to osteogenesis [30]. An upregulation of miR-330-5p observed in this study may indicate a loss in bone mass, which is supported by its positive correlation with OC. For miR-377-3p, existing literature indicates that it is a negative regulator of osteoclast formation/activation: Li et al. (2019) found that overexpression of miR-377-3p decreased RANKL, thus decreasing osteoclast differentiation [31]. According to Ji et al. (2022), miR-377-3p inhibits osteoclast activity [32].

Lastly, CTx (a marker of increased bone resorption and osteoporosis) was positively associated with miR-26b-5p and miR-145-5p, but negatively associated with miR-34c-3p. We found miR-26b-5p and miR-145-5p to be downregulated among depressed patients, which would lead to the assumption that there is a downregulation of CTx, and therefore also downregulated bone resorption. The potential role of miR-26b-5p has been highlighted above. Fukuda et al. (2015) noted that miR-145 is an inhibitor of osteoclastogenesis through core binding factor beta (Cbfb) and decreased bone regeneration in vivo [33]. However, the association of miR-145 with CTx was only significant and relevant when no controlling for additional factors was conducted, meaning that age, sex and/or BMI have an influence on the association of miR-145 with this bone turnover marker. The up-regulation of miR-34c-3p and its negative association with CTx suggests a decreased bone resorption activity. Unfortunately, the research on miR-34c-3p in relation to bone health is very limited. Eguchi et al. (2013) noted that miR-34c-3p is a possible osteocyte marker and repressor of osteoblast-maintaining genes, but more recent studies have not expanded this knowledge [34, 35]. A possible reason for the conflicting results in the current study compared to other papers could be due to the significant difference (*p = 0.026*) in CTx expression between the depressed and control groups.

Unfortunately, even though it was attempted to match the control group to the depressed group as well as possible, significant differences between groups in participant characteristics (age, BMI) were visible. Even though some associations of miRNAs with bone markers remained significant after controlling for age and sex, or BMI, age-, sex- or weight-related influences on bone could not be ruled out completely. Correcting for more of the confounding factors (e.g. alcohol consumption) could lead to an overcorrection, due to our limited sample size. Although in the current analysis a correction for age, sex and BMI was performed, a future study with a bigger sample size should try to match participants regarding these confounding variables. The notion could be raised that there are many variables involved which makes it difficult to clearly assess the relationship between bone health and depression. However, based on previous research already proving the association between depression and bone and by controlling for confounding variables, the risk of significant impact by external factors is minimized and the relationship between depression and bone health can be assessed in a relatively controlled manner.

Apart from individual miRNAs, this study identified the top 10 most enriched pathways regulated by miRNAs. Four signaling pathways are known to be related to depression pathogenesis and have been implicated in bone health/disease: the Forkhead box-O (FoxO) signaling pathway, the p53 signaling pathway, the Hippo signaling pathway as well as the Thyroid hormone signaling pathway.

FoxO proteins are classified as transcription factors [36]. The FoxO signaling pathway plays a role in the pathogenesis of depressive disorder [37]. BDNF, a mediator for FoxO, is commonly downregulated in depressed patients [37]. Furthermore, FoxO proteins (FoxO1, FoxO3 and FoxO4) are all expressed in bone cells, such as osteoblasts, osteoprogenitor cells, osteoclast progenitors, and osteoclasts, but not osteocytes [36]. A review from Ma et al. (2020) showed the functions of FoxO proteins in bone cells and found that these proteins have many but also conflicting functions in the regulation of bone metabolism. FoxOs have a function in the osteogenesis of osteoprogenitor cells, promote osteogenesis, and promote osteoblast functioning [36]. On the other hand, FoxOs have also been shown to suppress osteogenesis by restricting Wnt signaling in osteoblast progenitors [36]. Additionally, FoxOs regulate osteoclastogenesis and could therefore also impact bone resorption [36].

The second enriched pathway involved with depression is the p53 signaling pathway, which constitutes a network of genes which respond to stressors including DNA damage and play a role in DNA repair, cellular senescence, and cell cycle arrest [38]. Even though the results of the current study indicate a role of the p53 signaling in depression, the research regarding this pathway and depression is lacking. Regarding bone, a previous study by Yu et al. (2020) highlighted the role of p53 in the bone remodeling process and consequently in osteoporosis [39]. The study showed that a knockdown of p53 could partly reverse the decreases in BMD [39]. Furthermore, Han et al. (2022) showed that an upregulation of the CD137 gene inhibited osteogenic differentiation of BMSCs through the p53 Wnt/$\:{\upbeta\:}$-catenin pathway in mice [40]. Since 13 out of 19 miRNAs with altered expression among depressed patients in this study were involved in this pathway (Table 3), this could be a pathway connecting mental and bone health, but further research on the role of p53 signaling pathway in depression needs to be conducted to confirm or deny this assumption.

A third pathway involved with both depression and bone is the Hippo signaling pathway. This pathway encompasses certain molecular and cellular processes [41] involved with the pathophysiology of stress-related psychiatric disorders, such as depression [42]. The Hippo signaling pathway resides in the hippocampus, which plays a role in stress resilience [42]. The endpoints of the Hippo signaling pathway consist of two transcriptional co-activators: transcriptional factor with PDZ-binding motif (TAZ) and yes-associated protein (YAP) [42]. YAP is expressed in the midbrain and YAP/TAZ are regulated by the protein kinase complexes MST1/2 and LATS1/2. These complexes cause phosphorylation of YAP/TAZ. YAP/TAZ starts the transcriptional process of several genes regulating homeostasis, development and regeneration [42]. The HPA-axis releases glucocorticoids following stressful experiences, which in turn leads to elevated YAP protein levels and therefore increased transcription activity [42]. This highlights the association of the “stress”/HPA-axis, which is dysregulated in patients with MDD, with the Hippo signaling pathway. Other than its relationship to stress-related disorders, the Hippo signaling pathway also regulates bone metabolism, through YAP and TAZ [41]. These transcriptional factors play a part in the regulation of Runx2, Osx and Sox9, which regulate osteoblast differentiation, osteoblastogenesis and collagen type II expression [41].

Lastly, the Thyroid hormone signaling pathway, a pathway known to play a major role in depression pathogenesis, also plays a role in bone regulation. Thyroid hormones are significant components in the bone regulation process [43]. Mudri et al. (2023) highlighted the interplay between the Wnt pathway and thyroid hormones in bone metabolism, through an influence of Dickkopf 1 and Sclerostin (both part of the Wnt pathway) on bone resorption, inhibition of bone formation and a role in the maintaining process of bone, meaning that thyroid hormone levels and the Wnt pathway are connected and may cause changes in bone density [43].

In the following steps, the specific target genes of the 19 miRNAs were predicted. Seven target mRNAs/genes targeted by multiple of the differentially expressed miRNAs were detected. Not much is known about ABHD2, NUFIP2 and KLHL15 and their relation to bone. According to literature, TNRC6B plays a role in bone metabolism through the NOTCH and Wnt signaling pathways, and calcium modulating pathways [44, 45]. Regarding RAP2C, previous research has highlighted the potential of this target gene as a therapeutic target in Osteosarcoma, a type of bone cancer occurring mostly in children and adolescents, however the exact role RAP2C plays in bone-related pathways is not discussed [46]. Similarly to RAP2C, the role of WEE1 in Osteosarcoma has been researched and mentioned as a therapeutic target [47]. Lastly, heat shock proteins (HSP) could play important roles in bone pathologies [48]. Specifically, HSPA8 and its relation to Monoclonal nonspecific suppressor factor $\:{\upbeta\:}$ (MNSF$\:{\upbeta\:}$) plays a role in RANKL-induced osteoclastogenesis [48, 49]. Interestingly, HSPA8 was related to miRNAs (26b-5p, 106a-5p) which showed an association with bone formation markers in our data. An altered expression of these miRNAs will lead to a changed expression of HSPA8, and therefore osteoclastogenesis. Whether these alterations cause an increase or decrease of osteoclastogenesis should be further researched.

In this research a focus was put on miRNA research, even though other types of RNA such as messenger RNA and IncRNA, could also be of interest regarding the association between bone health and depression. MiRNAs are regulators of gene expression and research has shown that they are implicated in the pathophysiology of both bone health and depression, and therefore enable us to investigate the crossover between these conditions on a molecular level. Further, to analyze other types of RNA besides miRNA, different methods and resources are necessary, which were not part of the scope of the current study and should be focused on in future research.

The current paper has some limitations. Firstly, this study had a limited sample size (*n* = 20) due to the explorative nature of the study. This study reports an explorative analysis of miRNAs involved with depression, meaning that over 300 miRNAs were included for analysis. These types of explorative studies commonly have smaller sample sizes. Further, small sample sizes, here and in other studies, are consequences of the difficult recruitment of eligible patients in the mental health field. Additionally, the cohort included for analysis was controlled for many factors (e.g. comorbidities, medication) and the results did show statistically significant results despite the small sample size, indicating that the findings are robust. This pilot investigation provides a necessary basis for future studies with bigger sample sizes. Secondly, the sample differed between groups regarding age. This was due to the availability of participants for the study and has been aimed to correct for during analysis. Thirdly, BMD was measured among a sub-sample of the included depressed participants [8], but only a few of whom miRNA content could be measured had additional BMD outcomes and therefore BMD was excluded from analysis in the current study.

## Conclusion

**Fig. 3:**
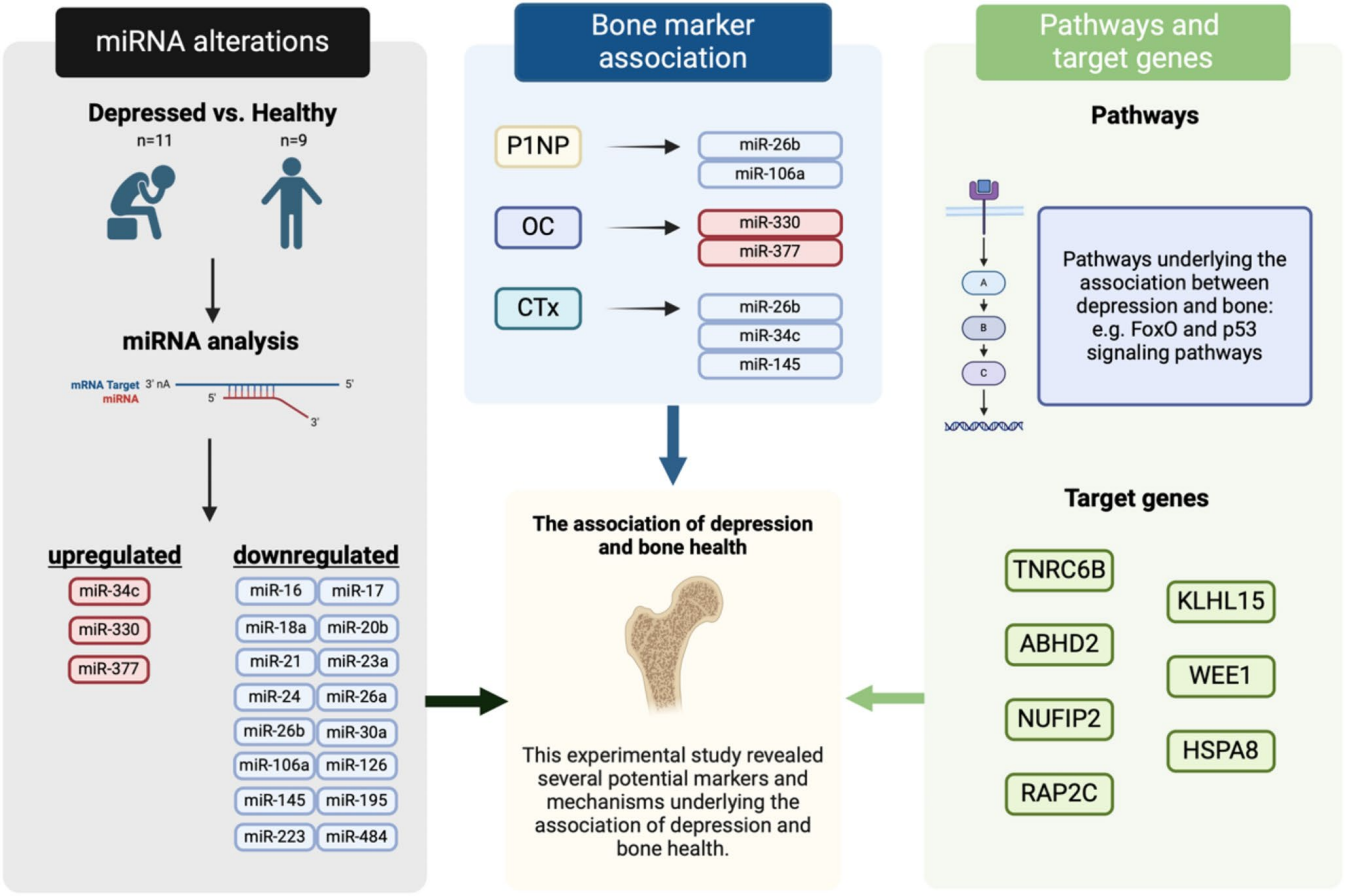
Graphical abstract representing the three aims in each column. On the left, miRNAs with altered expression among depressed patients is represented, whereas in the middle the associations of the three bone turnover markers with miRNAs are shown. On the right side, pathways and target genes predicted by the altered miRNAs are shown. These components combined all provide new knowledge regarding underlying mechanisms that influence bone health among depressed patients. Created in BioRender

In summary, the overall aim of the current study was to provide a better understanding of the potential underlying molecular and cellular mechanisms that play a role in the influence of depression on bone health. This study provided a further insight in the potential underlying mechanisms regarding the influence of depression on bone health (Fig. 3). Two miRNAs that showed the most potential to be mediators between the influence of depression on bone health and could possibly serve as biomarkers in the treatment of bone diseases among people with mental disorders were miR-26b-5p and miR-377-3p. Furthermore, genes (TNRC6B, HSPA8), and pathways (FoxO- and Hippo signaling pathway) were predicted to be relevant in the pathway regarding the influence of depression on bone metabolism.

## Electronic supplementary material

Below is the link to the electronic supplementary material.

Supplementary Material 1

## Data Availability

The data and materials supporting the findings of this study are available from the corresponding author SH, upon request.

## Abbreviations

ABHD2: Abhydrolase domain-containing protein 2
BDI-II: Beck Depression Inventory II
BDNF: Brain-derived neurotrophic factors
BMD: Bone mineral density
BMI: Body Mass Index
BMP: Bone Morphogenetic Protein
BMSC: Bone Marrow-Derived Stem Cells
CTx: Crosslaps
DNA: Deoxyribonucleic acid
DEXA: Dual-enery X-ray absorptiometry
EV: Extracellular vesicles
FDR: False Discovery Rate
FoxO: Forkhead box-O
HPA: Hypothalamic-Pituitary-Adrenal
HSPA8: Heat Shock Protein Family A
ICD: International Statistical Classification of Diseases and Related Health Problems
IGF-1: Insulin-like growth factor
KLHL15: Kelch Like Family Member 15
MDD: Major Depressive Disorder
NUFIP2: Nuclear FMR1 interacting protein 2
OC: Osteocalcin
OP: Osteoporosis
P1NP: Procollagen type 1 N-propeptide
P53: Tumor protein 53
RAP2C: Ras-related protein RAP-2c
RNA: Ribonucleic acid
TNRC6B: Trinucleotide Repeat Containing Adaptor 6B
WEE1: WEE1 G2 checkpoint Kinase
