# Supplementary material for: The underlying mechanisms of the association of bone health with depression – an experimental study
Source: Mol Biol Rep. 2025 Jan 27;52(1):163. doi: 10.1007/s11033-025-10230-x (PMC11772516; doi:10.1007/s11033-025-10230-x)
Supplement: Supplementary file 1 — Supplementary Material 1 [file 11033_2025_10230_MOESM1_ESM.pdf]

Supplementary Table 1: Linear regression of the 19 miRNAs with significant differences between the depressed vs healthy control group.

miRNA	N	Model 1		N	Model 2		N	Model 3	
		Regression value	***p-value***		***Adj. Regression value***	***p-value***		***Adj. Regression value***	***p-value***
**miRNA 16-5p**									
P1NP	19	1.713	0.302	19	1.693	0.272	19	0.925	0.576
OC	18	-0.241	0.613	18	-0.280	0.559	18	-0.241	0.560
CTx	19	0.008	0.645	19	0.009	0.605	19	-0.004	0.811
**miRNA 17-5p**									
P1NP	18	3.631	0.068	18	2.812	0.131	18	1.992	0.327
OC	17	-0.338	0.586	17	-0.585	0.336	17	-0.668	0.325
CTx	18	0.037	0.085	18	0.029	0.159	18	0.010	0.592
**miRNA 18a-5p**									
P1NP	15	2.568	0.157	15	1.306	0.467	15	2.078	0.313
OC	14	-0.384	0.486	14	-0.707	0.241	14	-0.420	0.507
CTx	15	0.017	0.366	15	0.003	0.871	15	0.006	0.769
**miRNA 20b-5p**									
P1NP	17	1.760	0.213	17	1.047	0.378	17	0.989	0.528
OC	16	-0.571	0.251	16	-0.823	0.083	16	-0.752	0.183
CTx	17	0.023	0.114	17	0.018	0.147	17	0.011	0.453
**miRNA 21-5p**									
P1NP	16	1.841	0.164	16	1.397	0.260	16	1.492	0.324
OC	15	-0.541	0.266	15	-0.769	0.130	15	-0.585	0.293
CTx	16	0.016	0.231	16	0.013	0.332	16	0.008	0.589
**miRNA 23a-3p**									
P1NP	18	1.692	0.092	18	1.182	0.200	18	1.082	0.337
OC	17	-0.249	0.470	17	-0.443	0.211	17	-0.366	0.358
CTx	18	0.016	0.161	18	0.011	0.310	18	0.005	0.696
**ümiRNA 24-3p**									
P1NP	17	1.265	0.101	17	1.164	0.084	17	0.786	0.335
OC	16	-0.272	0.329	16	-0.310	0.241	16	-0.375	0.226
CTx	17	0.012	0.202	17	0.011	0.215	17	0.004	0.686
**miRNA 26a-5p**									
P1NP	17	1.591	0.200	17	0.807	0.491	17	0.127	0.927
OC	16	-0.252	0.544	16	-0.507	0.238	16	-0.389	0.443
CTx	17	0.020	0.134	17	0.013	0.323	17	0.007	0.655
**miRNA 26b-5p**									
P1NP	16	10.224	0.005**	16	9.117	0.030*	16	9.589	0.034*
OC	15	1.201	0.214	15	0.792	0.483	15	1.425	0.257
CTx	16	0.101	0.015*	16	0.082	0.095	16	0.071	0.140
**miRNA 30a-5p**									
P1NP	16	2.363	0.141	16	1.748	0.256	16	0.679	0.687
OC	15	-0.318	0.539	15	-0.496	0.330	15	-0.493	0.418
CTx	16	0.029	0.098	16	0.025	0.158	16	0.014	0.459
**miRNA 34c-3p**									
P1NP	19	-8.269	0.123	19	-7.685	0.141	19	-4.729	0.429
OC	18	-0.211	0.894	18	-0.174	0.920	18	-0.011	0.995
CTx	19	-0.144	0.008**	19	-0.138	0.010*	19	-0.105	0.072
**miRNA 106a-5p**									
P1NP	19	1.981	0.044*	19	1.432	0.147	19	1.466	0.181
OC	18	-0.158	0.635	18	-0.388	0.276	18	-0.247	0.519
CTx	19	0.020	0.058	19	0.015	0.165	19	0.011	0.325

**miRNA 126-3p**									
P1NP	18	2.128	0.159	18	1.578	0.279	18	1.180	0.477
OC	17	-0.365	0.475	17	-0.601	0.246	17	-0.533	0.359
CTx	18	0.023	0.149	18	0.019	0.224	18	0.008	0.626
**miRNA 145-5p**									
P1NP	11	6.297	0.123	11	1.239	0.767	11	1.926	0.699
OC	10	-0.550	0.580	10	-1.968	0.107	10	-1.038	0.476
CTx	11	0.095	0.013*	11	0.060	0.168	11	0.081	0.100
**miRNA 195-5p**									
P1NP	19	3.103	0.134	19	2.434	0.213	19	2.065	0.331
OC	18	-0.213	0.719	18	-0.381	0.523	18	-0.316	0.629
CTx	19	0.023	0.316	19	0.017	0.452	19	0.006	0.794
**miRNA 223-3p**									
P1NP	17	1.103	0.183	17	0.871	0.296	17	0.785	0.405
OC	16	-0.253	0.410	16	-0.394	0.222	16	-0.307	0.384
CTx	17	0.013	0.155	17	0.011	0.225	17	0.006	0.542
**miRNA 330-5p**									
P1NP	18	-0.798	0.714	18	0.925	0.677	18	3.148	0.254
OC	17	1.101	0.061	17	1.774	0.002**	17	2.170	0.003**
CTx	18	-0.009	0.699	18	0.006	0.814	18	0.047	0.082
**miRNA 377-3p**									
P1NP	16	-0.292	0.624	16	-0.216	0.732	18	-0.142	0.830
OC	15	0.402	0.021*	15	0.443	0.019*	17	0.414	0.032*
CTx	16	0	0.987	16	0.000	0.970	18	0.004	0.559
**miRNA 484**									
P1NP	19	3.556	0.134	19	2.631	0.245	19	2.230	0.371
OC	18	-0.526	0.538	18	-0.885	0.330	18	-0.684	0.464
CTx	19	0.041	0.115	19	0.031	0.217	19	0.021	0.419

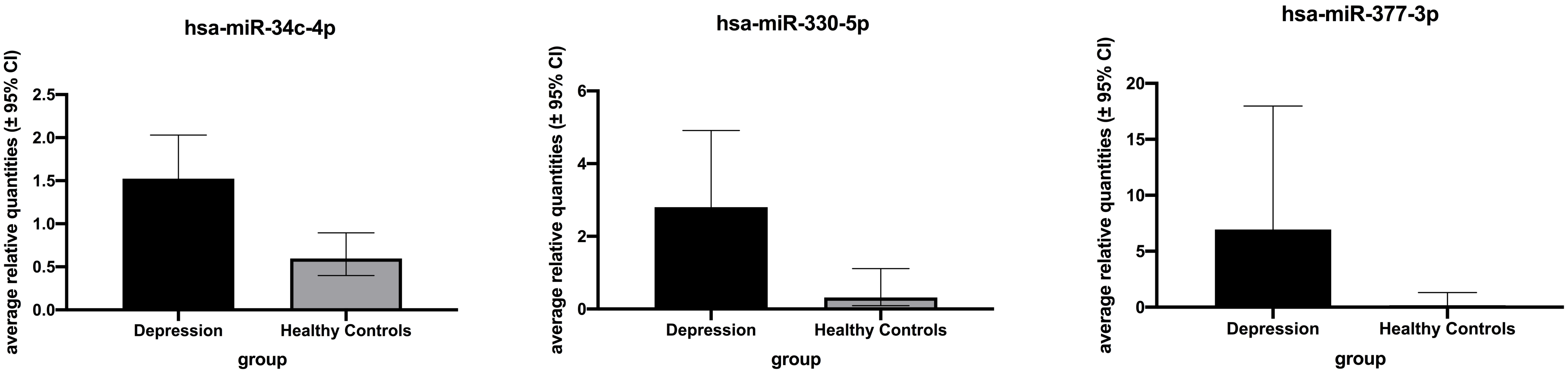

Supplementary Figure 1: Up-regulated miRNAs (N=3) in depressed patients compared to non-affected controls.

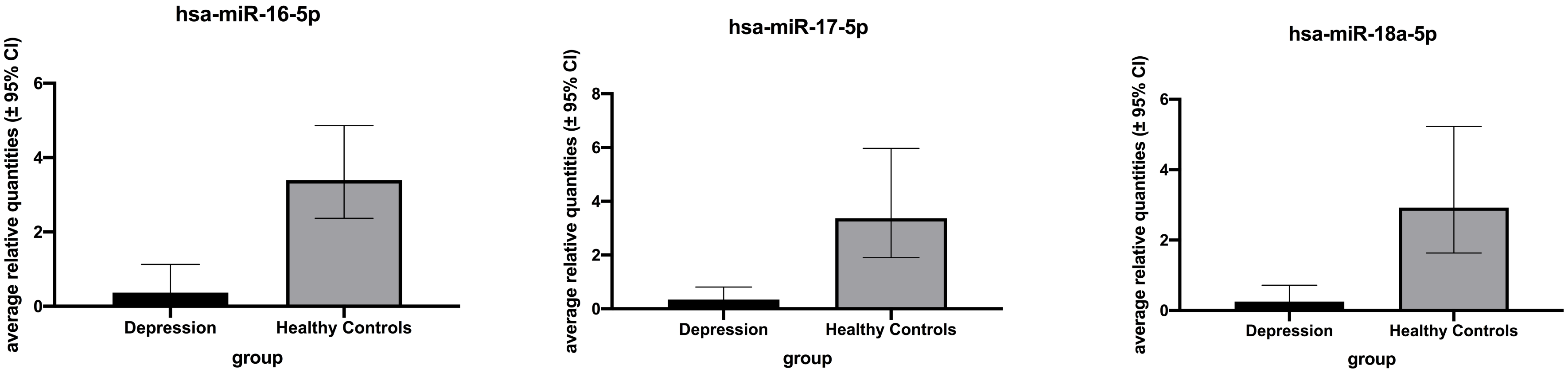

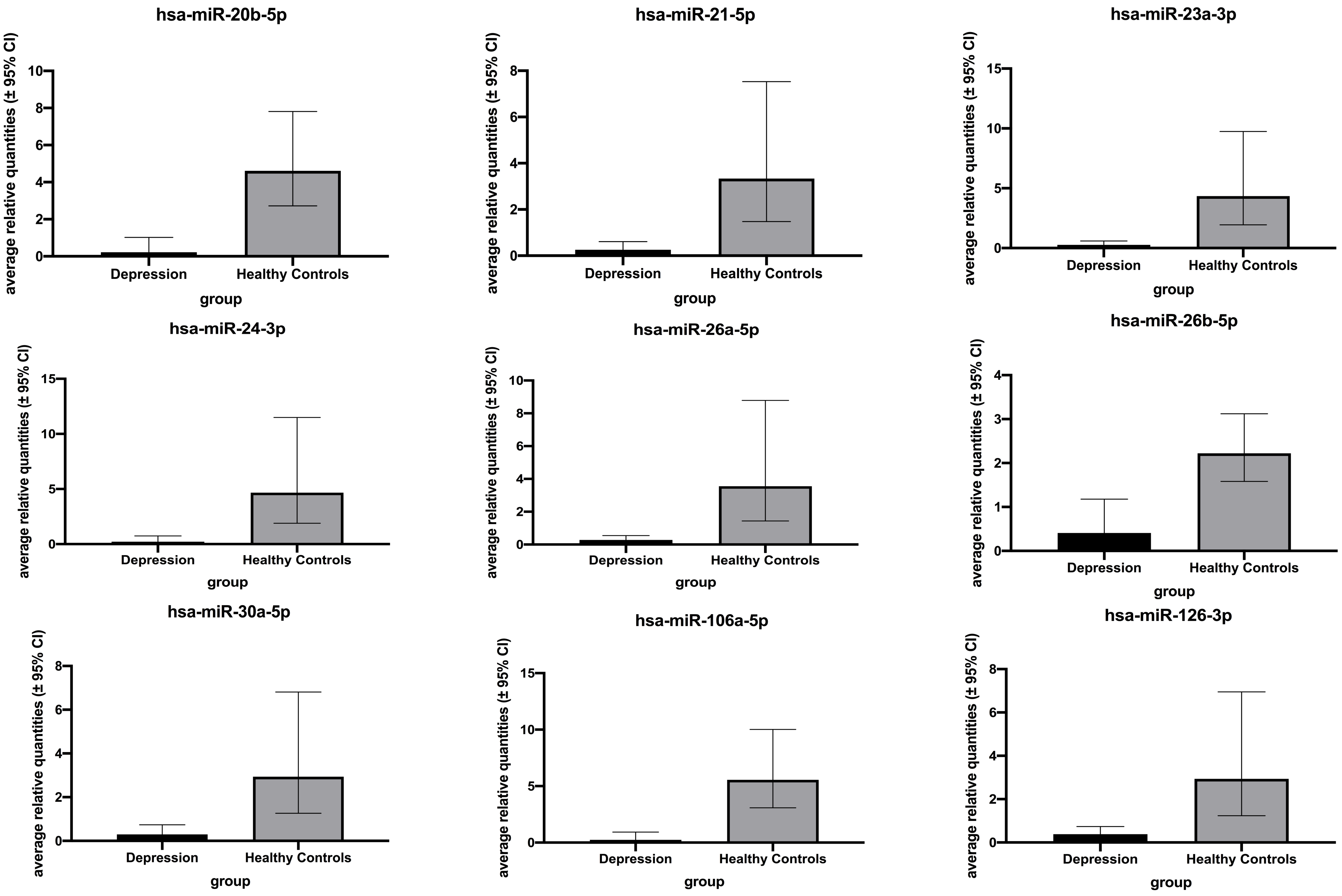
hsa-miR-20b-5p
hsa-miR-21-5p
hsa-miR-23a-3p
hsa-miR-24-3p
hsa-miR-26a-5p
hsa-miR-26b-5p
hsa-miR-30a-5p
hsa-miR-106a-5p
hsa-miR-126-3p
average relative quantities (± 95% CI)
Depression
Healthy Controls
group

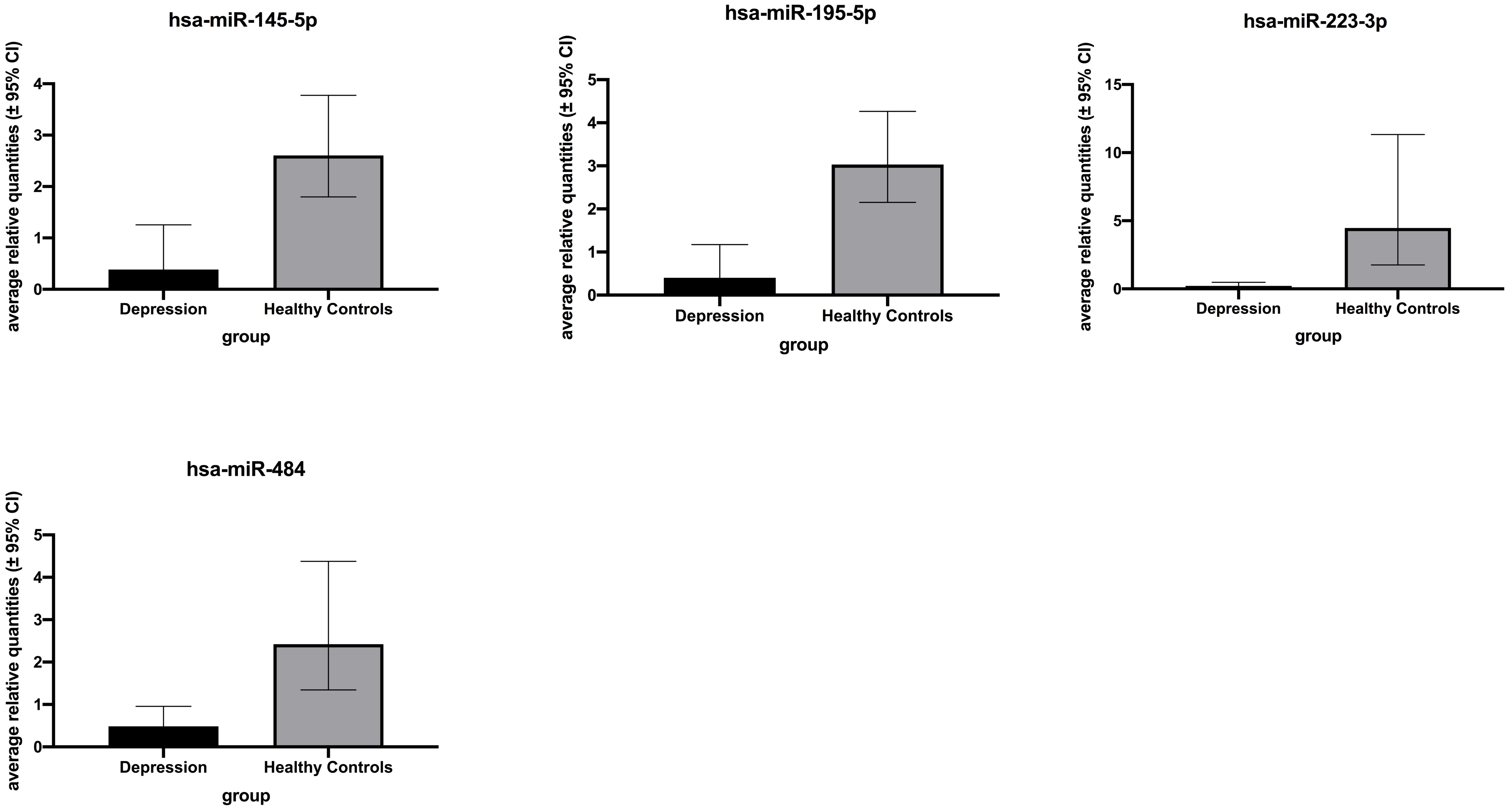

Supplementary Figure 2: Down-regulated miRNAs (N=16) in depressed patients compared to non-affected controls.

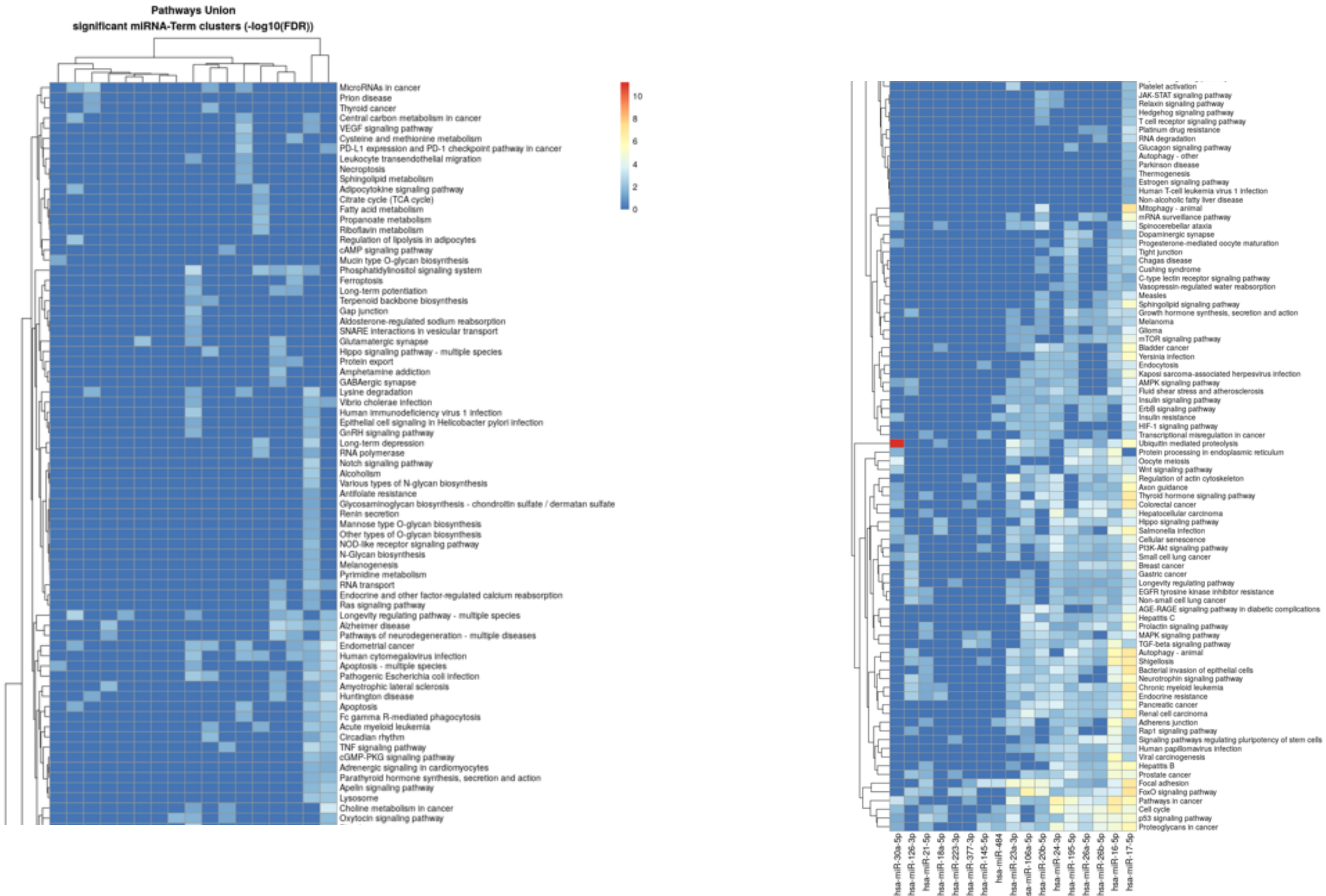

Supplementary Figure 3:. Enriched pathways identified by DIANA-miRPath 4.0 software

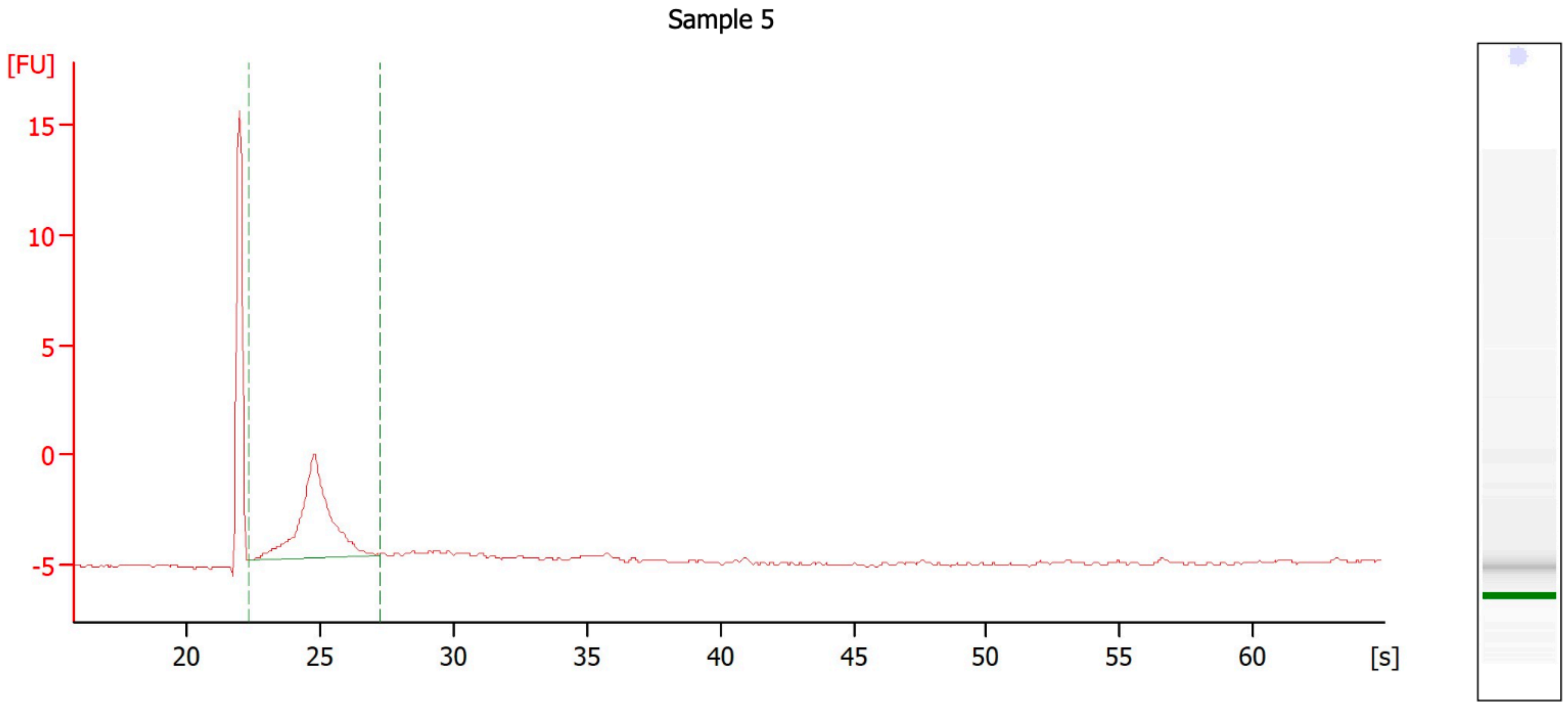

Overall Results for sample 5 : <u>**Sample 5**</u>

RNA Area:	10,1	RNA Integrity Number (RIN):	2.2 (B.02.11)
RNA Concentration:	27 pg/µl	Result Flagging Color:	
rRNA Ratio [28s / 18s]:	0,0	Result Flagging Label:	RIN: 2.20

Supplementary Figure 4: A typical Bioanalyzer profile (sample 5) of the EV miRNA samples used in the qPCR analysis.

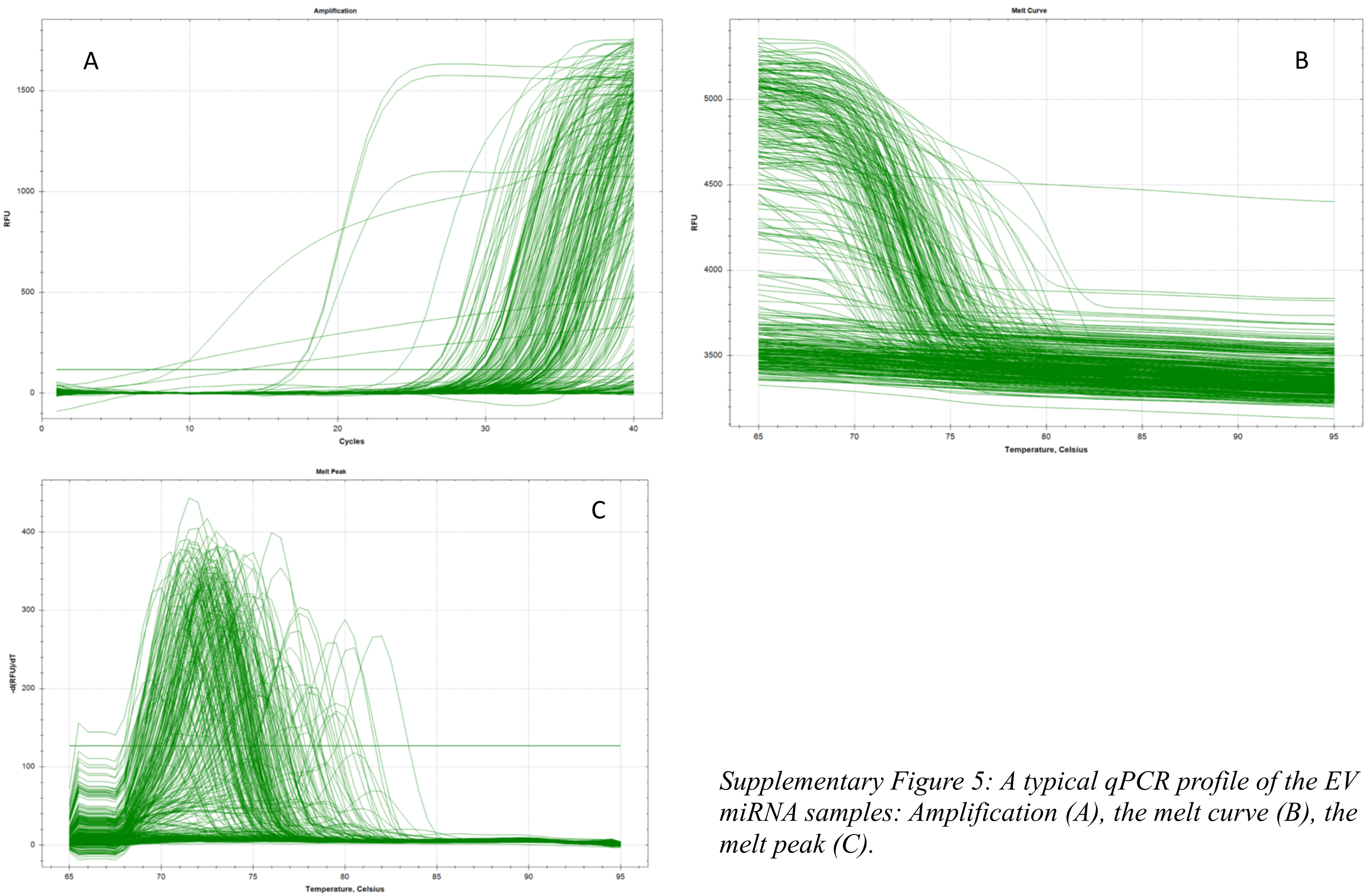

Supplementary Figure 5: A typical qPCR profile of the EV miRNA samples: Amplification (A), the melt curve (B), the melt peak (C).
